# A systematic review of the effects of psychiatric medications on social cognition

**DOI:** 10.1186/s12888-021-03545-z

**Published:** 2021-11-29

**Authors:** Zoë Haime, Andrew J. Watson, Nadia Crellin, Louise Marston, Eileen Joyce, Joanna Moncrieff

**Affiliations:** 1 Psychiatry Department, University College London, London, UK; 2 Institute of Neurology, UCL, London, UK; 3 Department of Primary Care and Population Health, UCL, London, UK

**Keywords:** Social cognition, Psychopharmacology, Schizophrenia, Antipsychotic, Benzodiazepine

## Abstract

**Introduction:**

Social cognition is an important area of mental functioning relevant to psychiatric disorders and social functioning, that may be affected by psychiatric drug treatments. The aim of this review was to investigate the effects of medications with sedative properties, on social cognition.

**Method:**

This systematic review included experimental and neuroimaging studies investigating drug effects on social cognition. Data quality was assessed using a modified Downs and Black checklist (Trac et al. CMAJ 188: E120-E129, 2016). The review used narrative synthesis to analyse the data.

**Results:**

40 papers were identified for inclusion, 11 papers investigating benzodiazepine effects, and 29 investigating antipsychotic effects, on social cognition.

Narrative synthesis showed that diazepam impairs healthy volunteer’s emotion recognition, with supporting neuroimaging studies showing benzodiazepines attenuate amygdala activity. Studies of antipsychotic effects on social cognition gave variable results. However, many of these studies were in patients already taking medication, and potential practice effects were identified due to short-term follow-ups.

**Conclusion:**

Healthy volunteer studies suggest that diazepam reduces emotional processing ability. The effects of benzodiazepines on other aspects of social cognition, as well as the effects of antipsychotics, remain unclear. Interpretations of the papers in this review were limited by variability in measures, small sample sizes, and lack of randomisation. More robust studies are necessary to evaluate the impact of these medications on social cognition.

**Supplementary Information:**

The online version contains supplementary material available at 10.1186/s12888-021-03545-z.

## Introduction (narrative synthesis element 1: theory development)

### What is social Cognition?

Social cognition is defined as the mental processes which underlie the ability to understand and act on the thoughts, intentions, and behaviours of others [1]. Deficits in social cognition have been found in psychiatric disorders including depression, schizophrenia and bipolar disorder [2–4] and can lead to the faulty interpretation of others’ intentions and thinking, as well as inaccuracies in identifying others’ emotions [5].

**Fig. 1:**
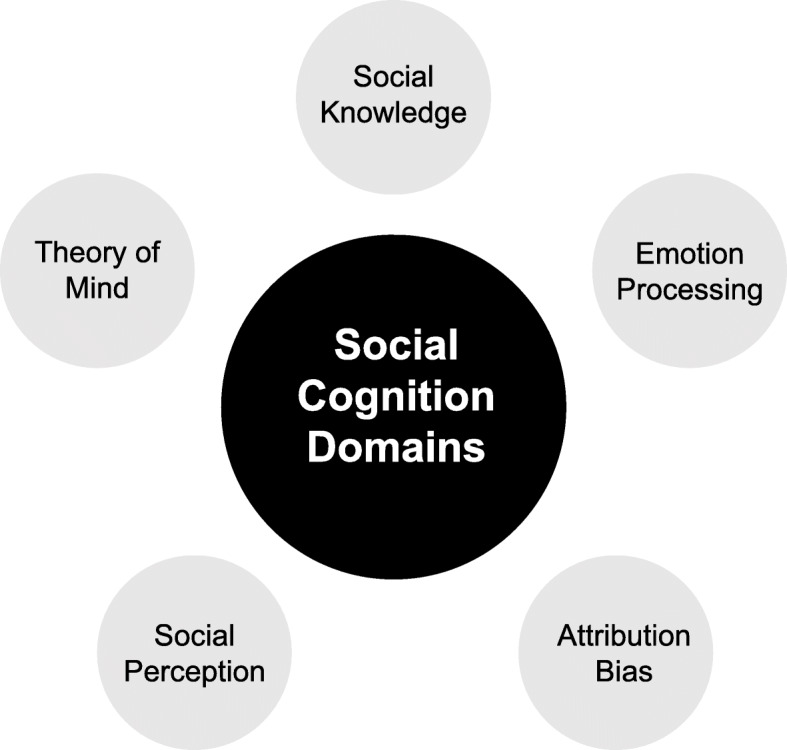
Social Cognition Domains identified by the NIMH

Social cognition can be separated into individual testable domains. However, many of these domains overlap, and there is no consistent agreement between cognitive scientists as to which are the most important. In psychiatry research the domains most frequently studied tend to reflect those identified by the National Institute of Mental Health (NIMH) at their meeting to define social cognition in schizophrenia in 2006 [6, 7]. These domains can be seen in Fig. 1, and include: Theory of mind (ToM) - the ability to ‘infer intentions, dispositions, and beliefs of others’ [8]**;** emotion processing - the ability to perceive emotions and interpret them appropriately [9]**;** social perception - the ability to process social cues and context to decipher social situations [10]; attributional bias - how people interpret the causes of events, which may be positive or negative in nature [11]; and social knowledge – how mental schemas of social situations guide behaviour [12]. Additional domains of social cognition tested in research include emotional intelligence, prejudice and stereotyping, and empathy [6].

Social functioning deficits are a core feature of most psychiatric diagnoses and considered an integral treatment target for many conditions, in order to promote recovery [13, 14]. Social cognition deficits have been associated with poor social functioning in several psychiatric disorders including schizophrenia, bipolar disorder, anorexia, Alzheimer’s disorder, and depression [2, 3, 15–17]. Additionally, in schizophrenia better social cognitive ability has been linked to better social functioning outcomes [18]. This makes social cognition a potential target for treatment interventions across psychiatric care.

### Psychiatric medications

The existing studies showing social cognition deficits in psychiatric disorders often involve people who are currently taking psychiatric medication [19]. However, it is unclear how these medications might affect social cognition. Many psychiatric medications have sedative effects, including benzodiazepines, antipsychotics (to varying degrees) and drugs used as mood stabilisers (22), and evidence suggests these drugs impair neurocognitive functioning in volunteers [20–23]. Antipsychotics also impair cognitive functioning in people with Alzheimer’s disease [24], but evidence on the effects of antipsychotics in people diagnosed with schizophrenia is inconclusive. Some evidence suggests that antipsychotics improve neurocognitive functioning [25, 26] and some that they impair it [27, 28].

Along with sedative effects, psychiatric medications affect emotion and motivation. Antipsychotics, for example, reduce motivation and suppress emotions in volunteers [29], effects which are also reported by patients [30], and these effects may impact on social cognition. On the other hand, psychiatric drugs may improve social cognition by alleviating symptoms that impair social interaction, such as psychotic symptoms and anxiety. Moreover, different agents within the same class may have different effects on social cognition, depending on their sedative profile and other effects [31].

Therefore, there is good reason to believe that psychiatric medications may influence social cognition, especially those with sedative actions that are known to impair neurocognitive functioning in volunteers. Clarifying these effects is important in order to understand the nature of social cognitive deficits in psychiatric disorders, and to evaluate the effects of treatment on social cognition and associated outcomes, such as social functioning. A previous review highlighted the paucity of evidence on the effects of antipsychotic treatment, but it did not explore the use of other medications or effects in volunteers [31]. Volunteer studies help to distinguish those effects that occur in the absence of symptoms of psychiatric disorders from those that are related to the disorder itself, or to the interaction of the treatment with the disorder. They can help with the interpretation of studies with patients who are taking medication, and ultimately improve our understanding of this complex area.

### Neuroimaging

The realisation that social disability may be linked to cognitive dysfunction has led to the employment of neuroimaging techniques to study this phenomenon in psychiatric populations, including the use of electroencephalography (EEG) and functional magnetic resonance imaging (fMRI). EEG can identify temporal changes in brain activity in response to specific tasks via event-related potentials (ERP), and fMRI is used to detect the location of changes in brain activity via variations in blood-oxygen-level-dependence (BOLD) [32, 33]. ERPs typically associated with social cognitive emotional stimuli are the P300-P400 potentials, where the brain shows activations 300-400 ms post-stimulus [34]. In fMRI, a social cognitive brain network has been identified and includes consistent activation of regions, including the temporo-parietal junction (TPJ), anterior cingulate cortex (ACC), superior temporal sulcus (STS), ventral and dorsal medial prefrontal cortex (VMPFC and DMPFC), precuneus, and inferior frontal gyrus (IFG) [35]. Neuroimaging studies investigating the effects of sedative medications on social cognition will help to identify any temporal or spatial neural changes in social cognitive brain regions, as a result of medication effects. This research is important in allowing researchers to assess the biological impact of psychiatric pharmaceutical treatments. In studies where patients have been using psychiatric medications with sedative effects long-term, permanent changes to structural and functional brain systems may inhibit the identification of medication effects on social cognition, Therefore, healthy volunteer and drug-naïve patient studies will be integral to our understanding of medication effects on social cognition in neuroimaging studies.

### Aims

Despite evidence of effects on neurocognitive functioning, there has been little consideration of how psychiatric medications affect social cognition. We hypothesised that psychiatric medications that produce sedative effects might affect social cognition, and we conducted a systematic review of the literature in this area. We included research on healthy volunteers as well as research conducted with patients with diagnosed psychiatric disorders.

An additional aim of this review was to explore any temporal or spatial brain differences between healthy volunteers and clinical populations with psychiatric diagnoses conducting social cognition tasks after administration of psychiatric medication using neuroimaging technology. Notable differences in brain activity may reflect the effects of medication on social cognitive processing.

The review will help to clarify the nature of any underlying deficits in social cognition in people diagnosed with psychiatric disorders, and this will help in the development of targeted treatments for social cognition, which may also improve social functioning and general outcomes [28].

## Method

### Protocol and registration

This review follows the PRISMA guidelines for reporting systematic reviews [36]. The review protocol is available on the PROSPERO registry, ID: CRD42018092883.

### Narrative synthesis

**Fig. 2:**
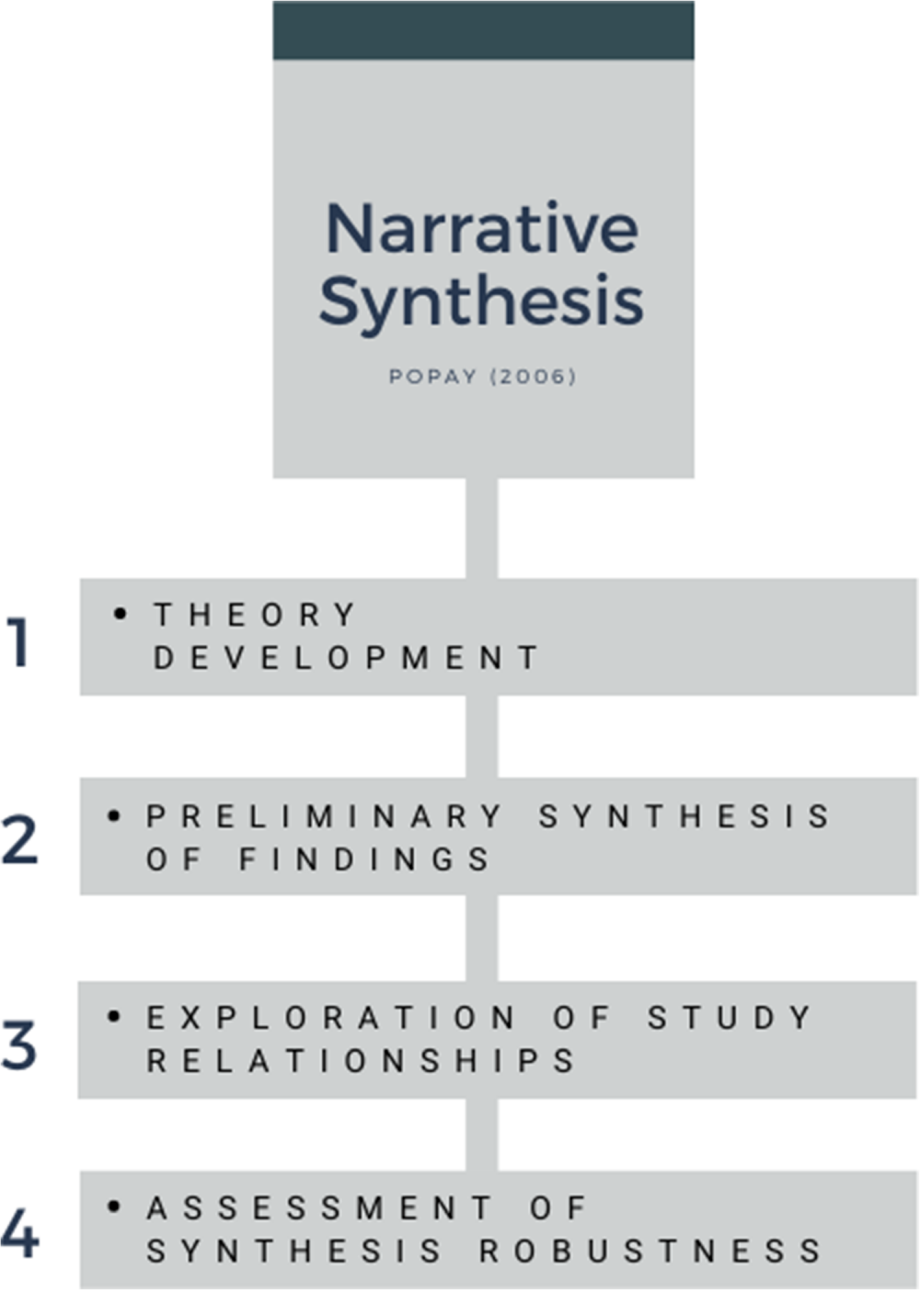
The four iterative elements of narrative synthesis [37]

The scope of our narrative synthesis was to examine the effects of sedative medications on social cognition. Following guidance from Popay et al. [37] we used four iterative elements shown in Fig. 2. As the first point suggests, we conducted an initial scoping of the literature to summarise the current research in the field, and in order to construct our search strategy. To address point two, we reported our findings in the results section and summarised relevant data from the included papers in a table (Table 1). In our discussion we critically explored relationships between the reported studies and went on to discuss the strengths and limitations of the current review, to address points three and four.

**Table 1:** Data Extraction of All Studies Included in the Narrative Synthesis, with Data Quality Scores

Author & Date.	Study Design	Sample	Medication Name/s	Dosage	Treatment Pre-Intervention	Placebo	Domain/s	Measure/s	Follow- Ups	Key Findings	Limitations	D&B Checklist Score
**Benzodiazepine Studies**												
Healthy Volunteers												
Blair and Curran (1999) [38]	Double-blind, independent group design	32 healthy volunteers	Diazepam	15 mg	N	Y	Emotion Processing	FERT	N/A	• Diazepam has a selective effect on the recognition of angry expressions. However, it did not affect the recognition of any of the other five expressions investigated.	• Limited sample size • Absence of a control group of psychiatric patients • No follow-up	12
Coupland et al. (2003) [39]	Randomised, counterbalanced, double-blind, placebo-controlled, within-subjects comparison	28 healthy volunteers	Diazepam	15 mg	N	Y	Emotion Processing	FERT	N/A	• Diazepam produced impairments in emotional recognition accuracy. The processing of surprise and disgust were most affected.	• No follow-up • Limited sample size	18
Murphy et al. (2008) [40]	Randomised, between-group, double-blind, placebo-controlled design	24 healthy volunteers	Diazepam	5 mg	N	Y	Emotion Processing	FERT	N/A	• No significant effect of Diazepam on accuracy or reaction times.	• Limited sample size • Low dosage of Diazepam	19
Pringle et al. (2016) [41]	Double-blind intervention	36 healthy volunteers	Diazepam	15 mg	N	Y	Emotion Processing	FERT	6, 7 or 8 days	• Diazepam makes participants significantly slower on emotional face recognition than controls.	• Limited sample size	19
Zangara et al. (2002) [42]	Double-blind independent group design	45 healthy volunteers	Diazepam Metropolol (selective antagonist of B1 adrenoceptors)	15 mg 50 mg	N	Y	Emotion Processing	FERT	N/A	• Diazepam impairs the ability to recognise angry and fearful expressions.	• No follow-up • Limited sample size	21
Nilsonne et al. (2018) [43]	Double-blind randomised controlled experiment.	Wave 1 = 37 healthy volunteers Wave 2 = 39 healthy volunteers	Oxazepam	25 mg	N	Y (Vitamin D3)	Empathy	Empathy for Pain Questionnaire	N/A	• No significant effect of Oxazepam on empathy	• Demographics of patient sample limits generalisability (all-male, largely university educated)	23
Patient Studies												
Zurowska et al. (2018) [44]	Intergroup Difference Study	The sample comprised 43 patients with schizophrenia in three groups: (1) during detoxification from benzodiazepines (*N* = 13), (2) after detoxification (*N* = 15), (3) a matched control group (*N* = 15).	Diazepam	concentrations of BZD differed significantly between patients	N	N	Emotion Processing/Empathy	Computerised emotion recognition task/Empathy Quotient	N/A	• Schizophrenia patients (during detox) addicted to benzodiazepines decreased ability to recognise emotions. Specifically, negative emotions (fear, sadness, and anger) compared to healthy volunteers	• Patients going through detoxification of bzds could be experiencing more severe symptoms than those addicted – may impact general emotional outcomes – no assessment of withdrawal symptoms • Small sample size • Did not control for anxiety and depression	12
Neuroimaging Studies (healthy volunteer and patient studies)												
Paulus et al. (2005) [45]	Double-blind, placebo-controlled, randomised dose-response study.	15 healthy volunteers	Lorazepam	0.25 or 1 mg	N	Y	Emotion Processing	Emotional Face Assessment Task - fMRI	N/A	• Lorazepam decreased activation in Amygdala and Insula when viewing emotional faces.	• No follow-up • Limited sample size	20
Olofsson et al. (2011) [39]	Double-blind experimental task.	45 healthy volunteers	Oxazepam	20 mg	N	Y	Emotion Processing	Affective Processing Task - EEG	1 week	• Oxazepam does not influence electrocortical indexes of emotional perception	• No patient sample • Only one medication type	14
Del-Ben et al. (2012) [46]	Randomised, balanced-order, double-blind, placebo-controlled crossover design	12 healthy volunteers	Diazepam	10 mg	N	Y	Emotion Processing	FERT	N/A	• Diazepam impaired the recognition of fear in female faces • Reduced activation in right Amygdala and right OFC • Reduced activation of bilateral ACC to angry faces • Enhanced activation of posterior left Insula	• Limited sample size • Patients may be aware of treatment arm	17
Richter et al. (2010) [47]	Double-blind independent group design	6 catatonic schizophrenia patients (recovered) 16 healthy controls (8 placebo/8 Lorazepam)	Lorazepam	A dose of lorazepam 1–2.5 mg was administrated intravenously 2–4 times (mean: 5.2 mg)	N	Y (saline)	Emotion Processing	IAPS - fMRI analysis	N/A	• High signal decreases in OFC and MPFC in catatonic patients during negative emotional stimulation after Lorazepam administration	• Limited sample size • Absence of a control group of psychiatric patients • fMRI measurements covered only the frontal lobe – so relationship between amygdala and MPFC regarding emotional processes remains unclear	18
**Antipsychotic Studies**												
Healthy Volunteers												
Lawrence et al. (2002) [48]	2 experimental test conditions (drug vs. placebo) - crossover study design - participants who took Sulpiride in week 1 testing took placebo in week 2 testing, and vice versa	14 healthy volunteers	Sulpiride	400 mg	Testing commenced 100 min following tablet (drug or placebo) ingestion in order to maximise drug levels during test administration. In order to provide an adequate washout period, two test sessions were separated by a median interval of 3 weeks. In each of the two testing sessions, participants completed a test of emotion recognition from the face and a control task of unfamiliar face matching (the Benton task).	Y (lactose)	Emotion Processing	FERT	baseline, ~ 3 weeks	• Following Sulpride use, recognition of anger facial expression at follow-up was impaired compared to baseline, other emotions intact	• Limited sample size • Short follow-up time	13
Rock et al. (2016) [49]	Between-subject, randomised, double-blind, placebo-controlled design	40 healthy volunteers	Quetiapine	150 mg	27 received Quetiapine for 7 days - dropout to *n* = 20 for Emotion Processing task	Y	Emotion Processing	FERT	baseline, one week	• No effect of Quetiapine on emotion processing ability in healthy participants at one week, compared to baseline	• No compliance measure • Healthy volunteers only • One-week duration only • Modest sample size • Dropout in Quetiapine arm (reduction of power) • Authors consultants for pharmaceutical company	22
Patient Vs. Healthy Volunteers												
**longitudinal studies**												
Behere et al. (2009) [50]	Short-term treatment follow-up	55 antipsychotic-naïve schizophrenia patients30 healthy controls	Risperidone	4 mg/daily	25 drug-naïve schizophrenia (DSM-IV) patients	N	Emotion Processing	TRENDS	Not specified (short-term)	• Schizophrenia patients showed impairments in emotion processing at baseline compared to healthy controls • Risperidone use in schizophrenia patients resulted in improvements in patient scores on the emotion processing task, when comparing their scores at baseline and follow-up	• Non-specified follow-up duration - may be practice effects • Only one antipsychotic type • Non-randomised design	16
Gaebel et al. (1992) [51]	Experimental task	23 schizophrenia patients 21 MDD 15 healthy volunteers	13 Perazine 10 Haloperidol (schizophrenia patients only)	The mean daily/cumulative dosages were 376/10160 mg CPZE and 445/16400 mg CPZE respectively.	11/23 schizophrenia patients’ drug-naïve, remaining 12 drug-free	N	Emotion Processing	FERT	baseline and 4 weeks	• Schizophrenia patients showed impairments in emotion processing at baseline compared to healthy controls • Both schizophrenia patient and healthy control groups improved at follow-up, larger improvements in schizophrenia patient group	• Pratice effects due to short follow-up time • Mixture of drug-naïve and drug-free patients	18
Olivier et al., (2015) [52]	Case-control design over 12 months.	92 FEP patients 100 healthy volunteers	Flupenthixol Decanoate (LAI)	10 mg	< 4 weeks of treatment (not a statistically significant difference at baseline, but difference is present)	N	Emotional Intelligence	MCCB	6-month, 12 month	• FEP performed significantly worse at baseline in all cognitive domains bar social cognition compared to healthy controls • FEP significantly improved in all MCCB domains (including social cognition) between baseline and 6 months. • No further improvements were seen in social cognition at 12 months in the FEP group, suggesting stability of emotional intelligence over time.	• Additional oral flupenthixol was prescribed at the discretion of the investigator • Not all patients were tested in their first language • Patients were not necessarily antipsychotic naïve • One antipsychotic type • FEP only	16
Zhou et al. (2017) [53]	12-week treatment study	56 schizophrenia inpatients 28 healthy volunteers	haloperidol (*n* = 12), fluphenazine (*n* = 8), chlorpromazine (*n* = 6), or trifluoperazine (*n* = 2). Risperidone (*n* = 28)	The mean chlorpromazine-equivalent dose was 502.0 ± 198.3 mg/d. The mean (±standard deviation) dose of risperidone was 4 ± 1.5 mg/d.	In the risperidone treatment group, 19 patients were drug-naive and 9 were drug-free (5 for at least 6 months and 4 for at least 1 month). In the typical antipsychotic treatment group, 17 patients were drug-naive and 11 were drug-free (8 for at least 6 months and 5 for at least 1 month).	N	Emotion Processing	FEDT	baseline, 4 weeks, 12 weeks	• Schizophrenia patients showed impairments in emotion processing at baseline compared to healthy controls • Risperidone improved social cognition in schizophrenia patients after 12 weeks compared to baseline, but not at 4 weeks.	• Mixture of drug-naïve and drug-free patients	18
Lewis et al. (1995) [54]	Experimental task	18 psychosis patients 10 healthy volunteers	Haloperidol	5-20 mg	Drug-free at baseline (for an unspecified time period)	N	Emotion Processing	FERT	baseline and 2 weeks	• Schizophrenia patients showed impairments at emotion processing at baseline compared to healthy controls • Haloperidol had no effect on patient performance at follow-up compared to baseline scores	• Small sample size • Short follow-up time period - practice effects • Did not subtype psychotic patients • Patients were not antipsychotic naïve	16
Wölwer et al. (1996) [55]	Experimental task	32 acute schizophrenia inpatients (S/a) 36 remitted schizophrenic patients (S/r) 21 healthy volunteers	Perazine Haloperidol Chlorpromazine Clozapine **S/r and S/a only**	The S/a were orally treated with either perazine (*n* = 20) or haloperidol (*n* = 12). The average daily dosage in chlorpromazine equivalents (CPZE) in the T0-T1 interval did not differ significantly (perazine: 436 + 217 mg CPZE; haloperidol: 531 + 313 mg CPZE). Among S/r 10 patients were treated with clozapine (mean daily dosage = 426 − + 144 mg CPZE), 21 received typical neuroleptic drugs either orally or as depot (mean daily dosage = 477 + 430 mg CPZE)	Among S/r 10 patients were treated with clozapine (mean daily dosage = 426 − + 144 mg CPZE), 21 received typical neuroleptic drugs either orally or as depot (mean daily dosage = 477 + 430 mg CPZE) and 5 patients were drug-free in the T0”-T1” interval. Five S/a, but none of the S/r, received anticholinergic medication.	N	Emotion Processing	FERT	baseline and 4 weeks	• Acute and remitted schizophrenic patients demonstrated a stable deficit in emotion recognition compared to healthy controls. • Antipsychotic medication had no effect on patient performance at follow-up compared to baseline scores.	• Non-randomised design • Short follow-up - practice effects • Patients were not antipsychotic naïve	15
Herbener et al. (2005) [56]	Short-term follow-up study	13 schizophrenia patients 13 healthy volunteers	Risperidone Ziprasidone Aripiprazole Haloperidol	mean dose R = 3.38 mg Z = 140 mg A = 30 mg H = 4.5 mg	< 4 weeks prior antipsychotic treatment in lifetime	N	Emotion Processing	CNB	baseline, average 31.3 days later (where clinically stable)	• Schizophrenia patients showed impairments at emotion processing at baseline compared to healthy controls• Antipsychotic medication had no effect on patient performance at follow-up compared to baseline scores	• Limited sample size • Non-randomised design • Short follow-up time - practice effects	11
Daros et al. (2014) [57]	Blocked experimental task	54 Healthy volunteers 29 Schizophrenia patients 28 Bipolar Disorder patients	**Schizophrenia** Risperidone (79.2%) Aripiprazole (12.5%) Haloperidol (8.3%) Ziprasidone (4.2%). **Bipolar Disorder** Risperidone (86.7%) Olanzapine (6.7%)	Drugs in chlorpromazine equivalents was 326.9 mg (SD = 218.9; range: 34.4–907.8 mg) for SCZ patients and 154.4 mg (SD = 125.7; range: 34.4–524.6 mg) for BP patients.	FEP patients. At study entry, some patients with SCZ and BP had previously been exposed to atypical antipsychotics (45.0%), antidepressants (30.0%), typical antipsychotics (15.0%), mood stabilizers/anticonvulsants (12.5%), and stimulants (12.5%), typically for brief periods of time in the months preceding their participation. No patient had taken a dose of any of these medications within three days of assessments, with the exception of BP (6.3%) and SCZ (12.5%) patients who were on maintenance antidepressant treatment started prior to study entry. Up to four weeks of prior cumulative lifetime antipsychotic treatment was allowed.	Y	Emotion Processing	CNB	baseline and an average of 6.8 weeks	• Schizophrenia patients showed impairments on emotion processing at baseline compared to healthy controls • Compared with controls, schizophrenia patients were worse at recognising mildly and moderately sad expressions at follow-up. • At follow-up, schizophrenia and bipolar disorder patients did not significantly differ from each other on any emotion category.	• Non-randomised design • Authors consult for pharmaceutical company	12
**cross-sectional studies**												
Kucharska-Pietura et al. (2012) [58]	Naturalistic treatment conditions	100 schizophrenia patients 50 healthy volunteers	Typical Atypical	Not stated	Twenty-eight (13 males) were treated with FGAs (perphenazine, *n* = 14; haloperidol, *n* = 14) and 56 (31 males) were treated with SGAs (olanzapine, *n* = 28; clozapine, *n* = 28). All patients were clinically stable after 3–4 weeks of antipsychotic treatment.	N	Emotion Processing Theory of Mind Empathy	FERT ‘Reading in the Minds Eye’ test Balanced Emotional Empathy Scale	N/A - cross-sectional	• Schizophrenia patients showed impairments at emotion processing at baseline compared to healthy controls • Antipsychotic medication had no effect on patient performance compared to healthy controls	• Non-randomised design • No follow-up evaluation • Antipsychotic medication not specified	15
Patient Only Studies												
**longitudinal studies**												
Kee et al. (1998) [59]	Baseline phase, brief placebo washout, and two double-blind phases 8 weeks double blind	18 schizophrenia patients	Haloperidol Risperidone	15 mg 6 mg	During baseline, patients received 15–30 mg/day of haloperidol for 3 weeks. This phase was followed by a period of 3–7 days of placebo wash-out. Upon entering the subsequent double-blind phases, patients first were randomly assigned to receive either 6 mg/day of risperidone or 15 mg/day of haloperidol for 4 weeks (fixed- dose phase). In the second double-blind phase, which also lasted for 4 weeks, medication doses from the previous phase could be changed according to symptom and side-effect considerations (flexible-dose phase).	N	Emotion Processing	FEIT	baseline and 8 weeks	• Risperidone improved the ability to perceive emotions compared to Haloperidol at follow-up compared to baseline	• Small sample size • Short follow-up time period - practice effects • Patients were not antipsychotic naïve	19
Harvey et al. (2006) [60]	8 week, multicentre, double-blind, parallel-designed, randomised, flexible-dose study	166 Schizophrenia patients	Risperidone Quetiapine	2-8 mg/daily 200-800 mg/daily	Sleep medication and benzodiazepines were allowed as needed but were not allowed within 24 h of clinical or neuropsychological assessments. Participants were taking antipsychotic medication at the start of the study and there was no titration period.	N	Emotion Processing	CNB	baseline, 8 weeks	• No significant differences associated with antipsychotic treatment at follow-up compared to baseline	• Supported by pharma company • Patients were not antipsychotic naïve • Short follow-up time period - practice effects • High drop-out at follow-up (%) – low generalisability	18
Mizrahi et al. (2007) [61]	Cross sectional study and a longitudinal study	17 FEP patients	Clozapine Risperidone Olanzapine Loxapine	Clozapine = 300 (n = 1) and 225 mg (*n* = 1) Risperidone = 4 mg (n = 4), 3 mg (*n* = 1), 3.5 mg (*n* = 1), or 1 mg (*n* = 1). Olanzapine = 10 mg (*n* = 4), 20 mg (n = 1), 15 mg (*n* = 1), or 2.5 mg (*n* = 1). Loxapine = 35 mg (*n* = 1)	Most subjects were started on atypical antipsychotic medications, except for two patients who were restarted on their previous clozapine dose (300 and 225 mg). The rest were started on risperidone 4 mg (*n* = 4), 3 mg (*n* = 1), 3.5 mg (*n* = 1), 1 mg (*n* = 1) or olanzapine 10 mg (*n* = 4), 20 mg (*n* = 1), 15 mg (*n* = 1), 2.5 mg (*n* = 1), and one patient was restarted on her previous 35 mg of loxapine.	N	ToM	Hinting Task	baseline - 6 weeks (measured every 2 weeks)	• Greatest improvement in ToM occurred during first 2 weeks of antipsychotic treatment, compared to baseline	• FEP patients only • Mixture of antipsychotic-naïve and drug-free patients • Non-randomised design • Short follow-up time period - practice effects	18
Sergi et al. (2007) [62]	8, week double blind, randomised study	73 outpatients with schizophrenia-spectrum disorder	Risperidone Olanzapine Haloperidol	4 mg 15 mg 8 mg	Patients were initially enrolled and tested at baseline on their pre-study medication; there was no medication washout period.	N	Emotion Processing/Social Perception	Half-profile of non-verbal sensitivity/IPT-15	baseline, 8 weeks	• No significant changes in social cognition associated with treatment over an 8-week study period.	•Pharmaceutical funding- medications for the study were provided by pharmaceutical companies • Modest group size and two random assignment paths - limited statistical power • Short follow-up time period - practice effects • Patients were not antipsychotic-naïve (no washout period)	21
Mizrahi et al. (2008) [63]	Cross sectional study and a longitudinal study	17 FEP patients	Typical Atypical	Not stated	The study was a cohort of consecutively admitted antipsychotic-free patients to the inpatient and outpatient Schizophrenia program who were willing to start antipsychotic medication. Patients had previously untreated psychosis and were antipsychotic-naïve at the beginning of the study, or had started or changed medication to improve symptoms in the previous 48 h.	N	Attribution Style	IPSAQ	baseline, 6 weeks	• Attributional style scores did not change during 6 weeks of antipsychotic treatment	• Small longitudinal cohort – may not have sufficient power • Short follow-up time period - practice effects • FEP patients only • Antipsychotic medication not specified	11
Fakra et al. (2009) [64]	Controlled, open, randomised and prospective design.	25 schizophrenia patients	Haloperidol Risperidone	Not stated	Followed a wash-out period of at least 1 week for prior antipsychotic treatment. Random assignment to Haloperidol or Risperidone treatment groups.Use of other antipsychotics or long-life benzodiazepines was prohibited. Benzodiazepines were not administered for a minimum of eight hours before emotional testing.	N	Emotion Processing	FEDT	baseline, 2 weeks, 4 weeks	• Greater beneficial effect of Risperidone than Haloperidol in schizophrenic patients’ ability to discriminate facial emotions at follow-up compared to baseline	• Small sample size • Patients were not antipsychotic-naïve • Short follow-up time period - practice effects	17
Penn et al. (2009) [65]	Random assignment to double-blind intervention	873 schizophrenia patients	Olanzapine Quetiapine Fumarate Risperidone Ziprasidone Perphenazine	(Zyprexa, Eli Lilly) (7.5 mg), (Seroquel, AstraZeneca) (200 mg) (Risperdal, Janssen Pharmaceuticals) (1.5 mg) (Trilafon, Schering-Plough) (8 mg) (Geodon, Pfizer) (40 mg)	Overlap in the administration of the antipsychotic agents that patients received before study entry was permitted for the first four weeks after randomization to allow a gradual transition to study medication. Concomitant medications were permitted throughout the trial, except for additional antipsychotic agents.	N	Emotion Processing	FEDT	baseline and 2 months	• Patients in all treatment groups (with the exception of Ziprasidone) showed small, non-significant improvements in emotion perception from baseline to two months	• Authors consult for pharma companies • Medications provided by pharma companies • Patients were not antipsychotic-naïve (medication was gradually titrated over 4 weeks following randomisation)	22
Roberts et al. (2010) [66]	Randomised, double-blind clinical trial.	223 Schizophrenia-spectrum patients	Olanzapine Quetiapine	olanzapine mean dose = 15.6 mg quetiapine mean dose = 455.8 mg *Chlorpromazine equivalents* of these doses are 312 mg/day and 607.7 mg/day, respectively.	Participants entered a 2-week titration period during which they were switched from their current medication to Olanzapine or Quetiapine.	N	Social Perception	SCRT	baseline and 6 months	• Olanzapine and Quetiapine significantly improve performance on 3/4 social cue recognition tasks at follow-up compared to baseline	• Patients were not antipsychotic-naïve (medication was titrated over 2 weeks following randomisation) • Pharmaceutical funding	20
Maat et al. (2013) [67]	8 week, randomised, multicentre, open-label study	48 schizophrenia patients	Aripiprazole Risperidone	maximum 30 mg maximum 6 mg	Overlap in the administration of the antipsychotic agents that patients received before study entry was permitted for the first 2 weeks after randomisation to allow for gradual transition. Concomitant medication other than antipsychotics was permitted throughout the trial; the dosage was restricted to a maximum of 30 mg diazepam or equivalent, 120 mg propranolol, and 12 mg biperiden or equivalent.	N	Emotion Processing	FERT	baseline, 8 weeks	• No significant effect of medication-group on endpoint performance on social cognition at follow-up compared to baseline	• High drop-out rate (few follow-ups) • Short follow-up time period - practice effects • Funded by pharma company • Patients were not antipsychotic-naïve	17
Shi et al. (2016) [68]	Single-arm, open-label study	95 Schizophrenia patients	Paliperidone	3-12 mg/daily	Single antipsychotic usage for at least 4 weeks before study.	N	Emotional Intelligence	MCCB	baseline, 6 months	• Treatment associated with improvements in 5/6 cognitive domains, but not social cognition	• Funding from pharma company • Open-label, single-arm design (efficacy bias)	19
Koshikawa et al. (2016) [69]	6 month pilot, open-label, randomised controlled study	21 Schizophrenia-spectrum patients	Paliperidone Palmitate Risperidone (LAI)	PP- doses of the drug were adjusted according to clinical status, upper limit of 50 mg /2 weekly. R (LAI)-The dose was determined depending on patient’s clinical status, with an upper limit of 150 mg/monthly	Inclusion: Having received risperidone long-acting injection for 2 months or longer. Exclusion: Current treatment with oral risperidone or oral palmitate risperidone. Current treatment with multiple oral antipsychotics.	N	Emotion Processing	SECT	baseline, 6 months	• No significant differences between the two groups in terms of the SECT accuracy at follow-up	• Small sample size • Patients were not antipsychotic-naïve (excluded if they were not currently being treated with antipsychotic medication)	21
Gultekin et al. (2017) [70]	Longitudinal naturalistic study	19 Schizophrenia-spectrum patients	Clozapine Risperidone	CPZE equivalent = 600 mg/day CPZE equivalent = 800 mg/day	being under current antipsychotic treatment included in inclusion criteria	N	Emotion Processing	FERT	baseline, 16–20 weeks	• Ability to recognise disgust faces poorer by a significant amount in the Risperidone group compared to the Clozapine group at baseline and significantly poorer after treatment with Risperidone then with Clozapine at follow-up. • Mean responses to facial emotions significantly shorter after Clozapine and Risperidone than at baseline	• Small sample size • Patients were not antipsychotic-naïve	16
**cross-sectional studies**												
Savina et al. (2007) [71]	Experimental task Naturalistic design	84 schizophrenia-spectrum patients 24 healthy volunteers	clozapine (*n* = 18) olanzapine (*n* = 20) risperidone (*n* = 23) perphenazine (*n* = 2) fluphenazine (*n* = 8) flupentixol (*n*=6) zuclopenthixol (*n* = 4) stelazine (*n* = 1) haloperidol (*n* = 2)	Not stated	received clozapine (*n* = 18), olanzapine (*n* = 20), risperidone (*n* = 23) or typicals (*n* = 23), including perphenazine (*n* = 2), fluphenazine (*n* = 8), flupentixol (*n* = 6), zuclopenthixol (*n* = 4), stelazine (*n* = 1) and haloperidol (*n* = 2), for at least 4 months. Most were also receiving mood stabilizers or other medications, but these were not systematically recorded. However, treating physicians were asked not to refer patients who received anticholinergic medication.	N	ToM	First-order Belief Task	N/A - cross-sectional	• Olanzapine and Clozapine groups performed similar to healthy controls on ToM task. • Risperidone and typical antipsychotic groups performed worse on ToM task (compared to healthy controls)	• Non-randomised design • No follow-up evaluation • Patients were not antipsychotic-naïve	13
Kucharska-Pietura et al. (2012) [72]	Naturalistic, pragmatic sample	84 Schizophrenia-spectrum patients	FGAs and SGAs	Not stated	39 patients were treated using conventional antipsychotic drugs (perphenazine, perazine, fluphenazine, haloperidol) and 61 were treated with atypical antipsychotic drugs (olanzapine, risperidone, amisulpride, clozapine and quetiapine). All patients were clinically stable after 4 weeks of antipsychotic use	N	Emotion Processing	FERT	N/A - cross-sectional	• No significant differences in performance between typical and atypical treatment groups.	• Non-randomised • No follow-up evaluation	15
Labuschagne et al. (2013) [73]	Experimental task	113 Early HD patients	Neuroleptics Not specified	Not stated	Of those taking neuroleptics (n1/429) almost all of the patients were on atypical neuroleptics except for one patient; the most common neuroleptic taken was olanzapine (14 patients). The neuroleptic daily dose range (expressed as the equivalent dose of chlorpromazine) was 50–800 mg. These patients may have been taking additional medications such as SSRI’s that were not fully listed. Adjusted for stage of disease	N	Emotion Processing	FERT	N/A	• In early HD neuroleptic use was associated with worse facial emotion recognition compared to those not using neuroleptics	• Emotion recognition deficits in HD may be due to facial perception impairments • Time constraints in testing – presenting only 10 stimuli per emotion • Single channel of emotion processing – faces only	13
Neuroimaging Studies (healthy volunteer and patient studies)												
Sumiyoshi et al. (2009) [74]	Longitudinal treatment design	20 outpatients with schizophrenia	Perospirone	Dose adjusted to optimise improvement in symptoms. Subjects who had already been treated with antipsychotic drugs, had medication switched stepwise to Perospirone monotherapy during the initial 6 weeks.	7/20 drug-free, 13/20 on antipsychotic medication	N	Social Perception	Script Tasks	baseline, 6 months	• Perospirone was associated with an increase in P300 ERP in the left PFC. • Performance on script tasks (social cognitive task) was improved during treatment, positively correlated with P300 changes.	• Subjects heterogeneous in terms of premedication • Small sample size due to large drop-out rate • Funding from pharmaceutical company	15
Takahashi et al. (2005) [75]	Single-blind, randomised, placebo-controlled design study.	13 healthy volunteers	Sultopride Fluoxetine (antidepressant)	25 mg 50 mg	N	Y (lactose)	Emotion Processing	Affective Processing Task - fMRI	Not specified 3 sessions	• After antipsychotic administration healthy volunteers showed decreased BOLD responses in limbic areas when viewing emotional stimuli	•Pharmacological actions may be on vascular and respiratory systems which in turn effect BOLD • Only healthy volunteers used • Pharmacological changes did not represent the minimal behavioural changes	14
Franken et al. (2008) [76]	Randomised, double-blind, placebo-controlled crossover design.	32 healthy volunteers	Bromocriptine (Beta-Blocker) Haloperidol	2.5 mg 2 mg	All subjects received a single oral dose of placebo (lactose), bromocriptine (2.5 mg), and haloperidol (2 mg) in a counterbalanced order. The medication was provided by the pharmacy of the Erasmus Medical Centre in indistinguishable capsules.	Y (lactose)	Emotion Processing	Affective Processing Task - EEG	weekly (for each condition −3 weeks total)	• Low dose haloperidol and bromocriptine did not change ERPs towards affective stimuli.	• Substantial dropout in Bromocriptine group – lower generalisability • Low doses of medication - due to unwanted side effects • Some participants received Domperidone to treat nausea	21

*Abbreviations*: *SMI* serious mental illness, *FERT* facial emotion recognition task, *fMRI* functional Magnetic Resonance Imaging, *EEG* Electroencephalography, *IAPS* International Affective Picture System, *MPFC* Medial Pre-Frontal Cortex, *OFC* Orbitofrontal Cortiex, *ACC* Anterior Cingulate Cortex, *FEIT* facial emotion identification test, *CNB* computerised neurocognitive battery, *ToM* theory of mind, *IPSAQ* internal, personal, and situational attributions questionnaire, *FEDT* facial emotional discrimination task, *SCRT* social cue recognition test, *FGA* first-generation antipsychotic, *SGA* second-generation antipsychotic, *MCCB* Matrics Consensus Cognitive Battery, *SECT* social emotional cognition task, *LAI* long-acting injection, *CPZE* chlorpromazine equivalent, *ERP* event-related potential, *TRENDS* tool for recognition of emotions in neuropsychiatric disorders, *DSM-IV* diagnostic statistical manual 4th edition, *BOLD* blood-oxygen level dependent, *D&B* Downs and Black Checklist

### Search strategy

We searched the following major databases: MEDLINE (OViD), Embase, Psychinfo, Web of Science, Lilacs, and Scopus as well as grey literature through greylit.org and opengrey.eu. Database-specific search terms included the keywords ‘social cognition’, ‘mental disorder’, ‘neuroleptic agents’, ‘sedatives’, and ‘tranquilisers’ with intervention-specific terms (including names of drug classes, and individual agents in classes that were not included as a whole, e.g., some sedative antidepressants), diagnosis-specific terms, outcome-specific subtypes and synonyms (see Additional file 1: **Appendix A** for full list of search terms and search strategy). An attempt to find additional studies was made through a backward reference search and contacting experts in the field.

### Inclusion/exclusion criteria

**Table:** 

Inclusion Criteria	Exclusion Criteria
Longitudinal or cross-sectional study designs	Published in a non-English language
Participants received or were being treated with a psychiatric medication with sedative properties ^a^	Qualitative, theoretical, or systematic review or meta-analysis papers
The population included healthy volunteers, humans with mental health or neuropsychiatric disorders	
Investigated a social cognition measure or task.	
Paper present in the initial search filtered for the following dates: From inception of database to 10th August 2019. Second search (on 30/12/2019): 10/08/2019–30/12/2019.	
Study present in grey literature searches on (greylit.org and opengrey.eu) and fit all the above inclusion criteria.	

^a^We included all antipsychotics, benzodiazepines, Z-drugs, and barbiturates. Tricyclic antidepressants, mirtazapine and trazadone were also included, and pregabalin. Drugs that are prescribed for mental disorders but predominantly used for physical health complaints, such as gabapentin and beta-blockers, were excluded

### Screening

Citations were imported to Mendeley and all duplicates were removed. ZH independently screened all citation titles for their applicability [77]. Titles that did not meet eligibility criteria were removed. Full-text papers were then screened and any uncertainties about inclusion were discussed with a second reviewer (AJW).

### Quality of assessment of studies

Study quality was evaluated using the Downs and Black checklist [78] as it allows for assessment of both randomised and non-randomised studies. The checklist evaluates papers on reporting, external validity, and internal validity (bias and confounding). It consists of 27 items scored with 0 points for ‘no/unable to determine’, or 1 point for a ‘yes’ response. Item 5 is scored differently with 0 points for a ‘no’ response, 1 point for a ‘partially’ response, or 2 points for a ‘yes’ response. The last item on the checklist regarding power was altered in concordance with a previous review conducted by Trac et al. [79] to rate whether a power analysis was calculated (1 point), or not (0 points). The maximum score for the checklist was 28, with the scoring ranges being (< 14) poor quality, (15–19) fair quality, (20–25) good quality, and (26–28) excellent quality.

## Results (narrative synthesis element 2: developing a preliminary synthesis)

### Search results

**Fig. 3:**
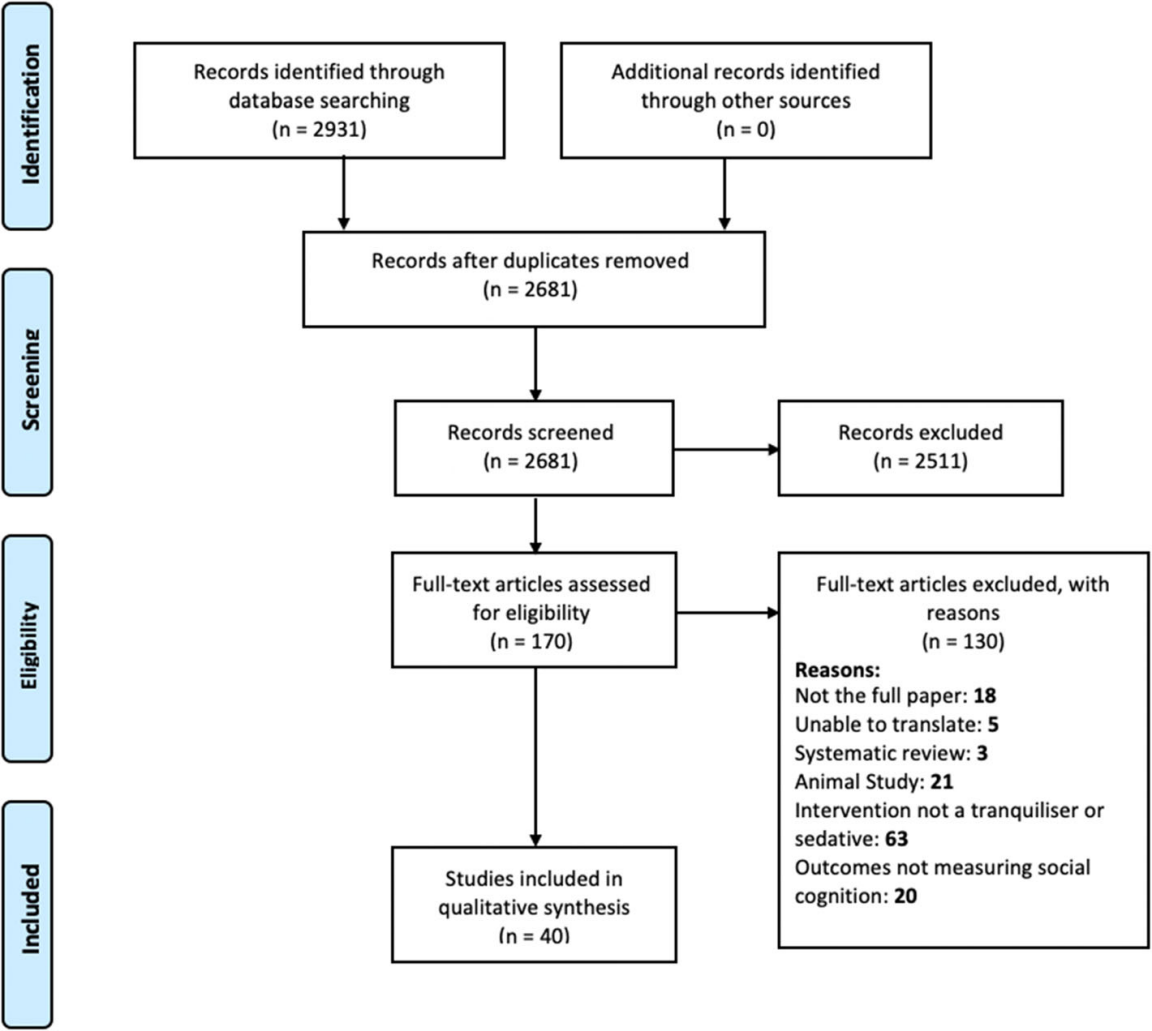
PRISMA flow diagram [36]

The search identified 2931 papers fitting the eligibility criteria, with 2681 remaining after de-duplication. The abstracts and titles of those records were then screened and 2511 were excluded due to not meeting the inclusion criteria. This resulted in 170 papers for full-paper screening. A further 130 papers were excluded during this stage, for reasons shown in Fig. 3. The remaining 40 full-text papers were used in the narrative synthesis. Data from these papers including study design, sample, medication (name, dosage), pre-intervention treatment, placebo (yes/no), social cognitive domains tested, social cognition measures, follow-up timepoints, key findings, and study limitations, were extracted and can be viewed in Table 1. Notably there were no studies of mood stabilisers, barbiturates, pregabalin or any sedative antidepressants.

### Quality of assessment of studies

Data quality was rated by ZH on all 40 full-text papers and AJW on 20% randomly selected papers. An interrater reliability score Cohens Kappa Coefficient of 0.85 was calculated [80], indicating good agreement between authors. Of the 40 included papers, 11 were rated poor and 20 were rated fair. Only 9 total papers were rated good, and none were rated excellent. Scores for each paper are reported in Table 1.

### Benzodiazepine studies

Four benzodiazepine studies conducted in healthy volunteers showed significant impairments in emotion recognition social cognition tasks following diazepam administration [39, 41, 44, 81], suggesting that sedative medication at a therapeutic dose impairs emotion processing. One of these papers also incidentally investigated the effects of Metropolol, a beta-blocker with mild sedative effects, and found no significant effect of the drug on emotion recognition [41]. A further study [42] showed a selective effect of diazepam on recognition of angry expressions only. This result may be due to using a small dose in comparison to other studies. One benzodiazepine study using oxazepam showed no effect on a measure of empathy [38].

One study was conducted in patients with schizophrenia, which looked at the effects of benzodiazepine withdrawal. Patients who were in the process of withdrawing from benzodiazepines were significantly impaired in recognising negative emotions compared to healthy volunteers, in contrast to patients who had already withdrawn, who were not. However, all patients were likely to have been on other medications [81].

### Neuroimaging studies of benzodiazepines and social cognition

All neuroimaging studies compared social cognition before and immediately after administration of the experimental drug. Del-Ben et al. [43] showed that a single dose of diazepam in healthy volunteers resulted in attenuated activation of the right amygdala when responding to fearful faces, although no evidence was found for this interaction when participants viewed angry faces. In another healthy volunteer study, Paulus et al. [82] showed that lorazepam attenuated activation in the amygdala and insula, and that the activation was significantly lower after 1 mg compared to 0.25 mg, suggesting a dose-dependent reaction in emotional processing regions. However, a study by Olofsson et al. [45] found no interaction between benzodiazepines and EEG activity during response to an affective processing task.

A study investigating benzodiazepine effects on patients with ‘catatonic’ schizophrenia and patients with bipolar disorder found that lorazepam induced BOLD signal decreases in the occipital cortex and medial prefrontal cortex (MPFC) in patients with schizophrenia when undertaking a negative emotion recognition task. This resulted in BOLD patterns resembling those of healthy volunteers taking a placebo drug during the same emotion recognition tasks [46]. However, at the time of the fMRI task all patients were taking either antipsychotic or antidepressant medications in addition to the administered lorazepam**.**

### Antipsychotic studies

#### Healthy volunteers

Only two studies tested the effects of antipsychotics on social cognition in healthy volunteers. A small crossover study by Lawrence et al. [47] (*N* = 14) found that recognition of angry facial expressions was reduced in participants taking sulpiride, but recognition of other expressions was not affected. In addition, a larger randomised parallel group trial of quetiapine versus placebo by Rock et al. [48] (*N* = 27) found no effect of the medication on facial expression recognition, though dropout rates were high (25%) in the quetiapine arm, which may have obscured an effect.

#### Patient studies

All studies comparing patients with schizophrenia and healthy volunteers found patients performed less well on social cognition tasks whether or not they were taking antipsychotics at baseline [49–54, 56, 61, 63, 68]. This included one study with patients who were drug naïve [61], two studies with patients who were drug-free at study commencement [53, 68], studies including participants with a mixture of drug-naïve, drug-free, and previous treatment for under 4 weeks [50–52, 54, 57, 72], and one study with patients stable on an antipsychotic [58]. Most longitudinal studies involving people with schizophrenia taking antipsychotics showed improvements in performance on social cognition tasks at follow-up compared to baseline [50–52, 55, 56, 59, 61, 63, 64, 66, 70], although some found no effect [49, 53, 54, 58, 60, 62, 65, 67] and one showed a decline [68].

When studies were classified by the prior medication status of participants, two longitudinal studies involved patients who were previously drug naïve. One of these studies detected improvements on an emotional processing task at follow-up [61], the other study involved an attributional style task, and found no effects of the medication [49]. Studies that involved patients who had a prior drug-free period, mostly found improvements in emotion processing tasks [50, 52, 53, 70], and one in a theory of mind task [56]. Some studies specified that participants were taking an antipsychotic at baseline prior to switching to another [51, 54, 55, 58–60, 62–65, 67, 68] and one did not describe the prior treatment status of participants [66]. In studies in which people were already taking antipsychotic treatment, results reflect effects of changing the type of antipsychotic rather than starting treatment.

One study tested healthy volunteers at baseline and follow-up to control for practice effects [50]. It found that patients with schizophrenia treated with antipsychotics (a mixture of people who were previously drug naïve (*n* = 11) or drug free (*n* = 12)) showed significant improvements in emotion recognition at 6 months compared to healthy volunteers.

One longitudinal study involving 29 people with schizophrenia and 28 with bipolar disorder explored dose-response relationships [68]. Findings showed that patients with schizophrenia who were taking higher doses of antipsychotic medication had more difficulty recognising sad and neutral facial expressions compared to those taking lower doses at follow-up. In bipolar patients, antipsychotic dose was unrelated to the accuracy of performance in judging emotions.

Studies comparing different antipsychotics produced inconsistent results. Some found that patients treated with second-generation drugs did better than those taking first-generation antipsychotics [55, 59, 66, 69, 70], but there was no consistent pattern to the results. Others found no difference between different agents or types of agent [57, 60, 62, 65, 67, 72]. The largest study by Penn et al. [64] showed improvements in all treatment groups (except for ziprasidone) on an emotion processing task, with no difference between individual second-generation drugs or between first- and second-generation drugs.

One study involving participants with Huntingdon’s disease showed poorer performance on facial recognition tests in those taking antipsychotics compared to those who were not, after controlling for the stage of the disease [71].

In this review, several studies were conducted by authors who received funding from pharmaceutical companies for research purposes or consulting. One study had a pharmaceutical company provide the medication for the research [65]. Studies that were conducted by authors who received pharmaceutical company funding found either improvements in social cognition after antipsychotic administration [55, 64, 73], or no effect of the drug on performance [60, 63, 65]. However, improvements were also shown in studies that did not rely on pharmaceutical funding [50–52, 56, 61, 66].

#### Neuroimaging studies of patients and social cognition

A study by Sumiyoshi et al. [73] investigated the effect of the antipsychotic, perospirone, on social perception in schizophrenia patients. They found an increase in the P300 ERP activation in the left pre-frontal cortex (PFC), as well as improvements in the social cognitive script task, after 6 months treatment compared to baseline.

A study investigating the effect of sultopride on emotion processing in healthy volunteers found decreased BOLD responses in the amygdala when viewing negatively valenced stimuli compared to before sultopride administration [74]. There was also increased activation in the PFC identified during positron emission tomography (PET) scans. However, behaviourally they found minimal changes to performance on social cognition tasks. Additionally, a crossover EEG study by Franken et al. [75] with healthy volunteers, found that both the dopamine agonist bromocriptine, and antipsychotic haloperidol produced no significant difference in emotion-related ERPs (P300-P400) compared to before drug administration. This study used low doses of medication, however, and some participants were also prescribed domperidone to treat nausea.

## Discussion (narrative synthesis element 3: exploring the relationships within and between the studies)

Clarifying the effects of prescribed medication on social cognitive ability is important since social cognition appears to be impaired in people across psychiatric diagnoses, and this impairment may be related to deficits in social functioning that represent a significant disability. Hypothetically, psychiatric drugs may impair social cognition due to their sedative effects, or may, through improving psychiatric symptoms, benefit social cognition.

The findings suggest that psychiatric drugs with sedative properties, such as benzodiazepines, can impair emotion recognition in healthy volunteers [39, 41, 42, 44, 81]. Findings were most consistent for emotion processing following the use of diazepam, however few studies were conducted using other benzodiazepines or measures of social cognition. Two neuroimaging studies investigating lorazepam found decreased activation in the social cognitive neural network during emotion processing [46, 82]. These findings suggest sedative effects of lorazepam may be altering brain processes required for emotion recognition, although neither study used a behavioural measure to confirm the effects on social cognitive ability. In contrast effects of antipsychotics on healthy volunteers were inconsistent, but only two studies were identified. As antipsychotics have different pharmacological profiles and cause varying levels of sedation, different agents may have different effects. Further research is required to clarify effects of antipsychotics on social cognition in volunteers, especially considering the evidence that antipsychotics impair neurocognitive performance and their reported effects on emotional reactivity.

Results of studies with patient populations found that antipsychotic treatment improves or has no effect on social cognition in patients with schizophrenia. The studies suffered from several important methodological limitations, however. First, practice effects in cognitive tasks are common [76], and as most studies in this review had short follow-up time windows (averaging 3.2 months) it is expected that improvements would be caused by task memory from earlier sessions. Only one study controlled for practice effects by including a healthy volunteer control group. The study identified practice effects, but also showed an additional improvement in social cognition that was independent of practice effects [51]. Second, studies did not distinguish the effects of the medication from the effects of changes in symptoms. Symptom improvement may occur as a result of antipsychotic treatment but may also occur spontaneously. One of the present studies detected a correlation between psychotic symptoms and social cognition [53], but ultimately, placebo-controlled comparisons are needed to reliably detect treatment-specific effects.

In contrast to studies showing improvement in social cognition with antipsychotics, one study on emotion processing identified a negative effect with a dose-response relationship, such that higher doses of antipsychotics related to higher levels of social cognitive impairment in patients with schizophrenia, but this was only found in people diagnosed with schizophrenia and not with bipolar disorder. The study with patients with Huntingdon’s disease also found worse facial recognition performance associated with antipsychotic use, even after adjusting for disease severity [71]. This is consistent with the evidence of reduced neurocognitive functioning in people with Alzheimer’s following antipsychotic use, but further studies are required to clarify the effects of drugs in other psychiatric conditions.

Patients who experience psychiatric disorders are likely to experience neurocognitive deficits such as poor attention and decision-making skills due to the nature of their symptoms [83], which may directly prevent successful social cognitive ability [27]. In addition to this, some research has found that patients with a mental health diagnosis are more likely to have poorer intellectual abilities than the healthy population [84], which could result in difficulties with language and communication skills. These difficulties may make individuals less experienced or confident in a social environment and have a negative influence on social cognition as a result. The studies examined here confirmed that there is an impairment of social cognition in people experiencing a psychotic episode, even before drug treatment is started. However, the research base is currently not adequate to unravel whether there are additional positive or negative effects associated with the use of psychiatric drugs.

Neuroimaging findings suggest that medication may be affecting brain processes that have been found to be associated with social cognitive ability. Sumiyoshi et al. [73] found an increase in the P300 ERP during a social perception task in patients with schizophrenia after antipsychotic administration, which was positively correlated with their task performance. However, only 7 of 20 participants started the study drug-free, and 8 participants dropped out after the baseline assessment, making it difficult to make firm conclusions. Takahashi et al’s [74] study on an affective processing task showed decreased BOLD responses in the amygdala and greater activations in the PFC following antipsychotic administration in healthy volunteers. This was noteworthy as the PFC is known to attenuate amygdala activation during emotional processing [85]. Therefore, it is possible antipsychotic medication is working directly on the PFC, and decreased amygdala signals are secondary to this.

### Strengths and limitations (narrative synthesis element 4: assessment of the robustness of the synthesis)

One of the most important strengths of this review was establishing the current literature on the effects of sedative psychiatric medications on social cognition using a rigorous search strategy of published and unpublished work.

We included all psychiatric populations, and healthy volunteer studies in our search. However, in our review we found the research was largely limited to studies of benzodiazepine effects in healthy volunteers, and studies of antipsychotics in patients with schizophrenia with one study of patients with the neuropsychiatric disorder, Huntingdon’s disease [86]. Research on neurocognitive function suggests that antipsychotics, in particular, may have specifically detrimental effects in people with psychiatric disorders, such as Alzheimer’s, and further research on their effects on social cognition in people with these disorders would be valuable.

We made efforts to also include all prescribed psychiatric medications with sedative effects in our search, but we may have omitted some medications that are not commonly used. We also excluded drugs that are prescribed for mental disorders but are predominantly used for non-psychiatric indications, such as gabapentin or beta-blockers. We also did not include drugs with sedative effects that are routinely used for physical disorders, such as opiate anaesthetics, for example, and we also did not include recreational sedatives such as alcohol or heroin in our search. The review focused on prescribed psychiatric medication in order to clarify the effects of these medications in people with diagnoses of mental disorders, but recreational drugs are commonly used amongst patients with a mental health diagnosis [87], and their sedative effects may also influence social cognitive ability. Therefore, this should be an important consideration for future research in the area.

An integral strength of our search for this review was the inclusion of all known terminology for social cognitive domains and measures. However, this was difficult due to the use of interchangeable terms for similar items, exposing a feature of the social cognition field that needs to be addressed.

A limitation of the current review was the poor quality of available studies. Our data quality analysis tool allowed us to identify several deficiencies with current papers available in the field, such as small sample sizes, non-randomised designs, and few adherence to medication measures. Only four of the studies found conducted power analysis to qualify their sample size. This resulted in many of the included studies having small numbers of recruits, undermining the internal and external validity of the research findings. During quality analysis, researchers also found only three of the longitudinal studies included were recording medication adherence. In addition to this, very few studies considered the influence of practice effects, which have an important influence on the results of longitudinal studies of cognitive performance, and there were few randomised placebo-controlled studies that would allow conclusions about whether changes in patients taking antipsychotics were attributable to specific medication effects, or whether they were the result of unrelated symptom improvement or of practice effects. Additionally, only one study of antipsychotics, and no benzodiazepine studies, looked at dose-dependent effects. This variability in studies also restricted analysis of the papers included, making a meaningful meta-analysis impossible to conduct.

One other major limitation of this review was that 80% of the included studies explored emotion processing tasks, leaving the other domains of social cognition largely ignored in the literature. Resultingly, our review is more of an insight into the effects of sedative medications on emotion processing, rather than the broader area of social cognition as a whole.

Finally, benzodiazepines have reasonably consistent effects, but antipsychotics vary widely in their receptor targets, pharmacological actions and sedative profiles [47, 48]. The studies examined did not enable a comprehensive comparison of the effects of different agents within any class of drugs. In addition, no studies were found that assessed effects of other prescribed psychiatric drugs with sedative properties, such as mood stabilisers, sedative antidepressants or pregabalin.

### Further research

We suggest that further research of higher quality is needed to clarify the effects of sedative medications on social cognition in healthy volunteers and patients with psychiatric diagnoses.

Further studies conducted with neuroimaging techniques will allow better insight into structural or functional brain changes resulting from administration of psychiatric medication with sedative effects. Conducting these studies with a behavioural performance element will also allow researchers to identify if brain changes are consistent with changes in social cognitive ability.

Further studies also need to control for practice effects, and studies involving patients should include placebo or no treatment control groups in order to distinguish the effects of medication from the natural evolution of psychiatric symptoms. Studies should be conducted across a range of social cognition domains, to ensure we are getting an accurate picture of complete social cognitive ability. Additionally, studies should be conducted across a range of psychiatric medications with sedative properties, to ensure we are able to identify any significant differences between drugs, and in different psychiatric diagnoses to clarify the effects of medication across conditions.

Notably, recent research in the field of social cognition and psychiatry has focused on the potential benefits of non- pharmacological interventions, such as social cognition and interaction training (SCIT), metacognitive reflection and insight therapy (MRIT), and metacognitive training. A review in 2009 by Choi and colleagues [88] found that five intervention studies showed promising results for social cognitive improvements in patients with schizophrenia, and a comprehensive review by Kurtz et al. [89] showed large effect sizes for training on facial affect recognition, moderate effect sizes on theory of mind, and small effect sizes on attribution bias for patients with schizophrenia. Although some studies included in these reviews used control groups, the majority of studies failed to control for potential medication effects in participants with schizophrenia. Psychiatric medication use alongside a social cognitive training intervention may cause improvements or deficits in participant outcomes, which we consider an important clinical implication in treatment implementation. Therefore, we suggest future research in this area accounts for psychiatric medication use in the analysis of intervention effectiveness.

## Conclusion

Deficits in social cognition have been identified in people with psychiatric diagnoses, and are associated with impaired social functioning, yet we remain uncertain to what extent these are attributable to the effects of the disorder or the effects of its treatment. A number of healthy volunteer studies suggest that diazepam and lorazepam can impair emotion processing abilities. Studies on antipsychotics were inconclusive and suffered from methodological limitations. There were no studies on any other drugs with recognised sedative properties, and studies focused mainly on the emotion processing domain of social cognition. Better data on the ability of drugs to affect social cognition will help to improve our understanding of the nature of social cognitive deficits in mental disorders, and the effects of treatment. Optimising the treatment of social cognition could potentially lead to better social functioning outcomes.

## Supplementary Information

**Additional file 1: Appendix A**. Literature Search Strategy.

## Data Availability

Not Applicable.
