# Supplementary material for: A systematic review of the effects of psychiatric medications on social cognition
Source: BMC Psychiatry. 2021 Nov 29;21:597. doi: 10.1186/s12888-021-03545-z (PMC8628466; doi:10.1186/s12888-021-03545-z)
Supplement: Supplementary file 1 — Additional file 1: Appendix A. Literature Search Strategy. [file 12888_2021_3545_MOESM1_ESM.docx]

**Appendix A: Literature Search Strategy**

The following databases were searched:

| **Database Name** | Results Retrieved |
| --- | --- |
| **MEDLINE** | 207 |
| **Embase** | 919 |
| **PubMed** | 311 |
| **CINAHL** | 0 |
| **LILACS** | 0 |
| **Web of Science** | 1259 |
| **PSYCINFO (+ PsycExtra)** | 229 |
| **SCOPUS** | 0 |
| **Greylit.org** | 0 |
| **OpenGrey.eu** | 6 |

The search strategy was to combine searches of:

- “tranquiliser” and “sedative” and “antipsychotic” and “benzodiazepine” and “z-drug” and “barbiturate” and “tricyclic antidepressant” and “Mirtazapine” and “Trazadone” related terms. Where MeSH terms did not include specific medication names, these were listed separately.
- “social cognition” related terms
- “psychosis” and “psychiatric disorder” and “healthy volunteer”. Where MeSH terms did not include specific psychiatric diagnosis, these were listed separately.

**Example Search:**

MEDLINE was searched using the OvidSP interface on 30/12/2019 from inception to present dates.

| Step | Search Terms | Results Retrieved |
| --- | --- | --- |
| 1 | (anti-anxiety agents or antipsychotic agents or psychotropic or antipsychotic* or Hypnotics or Sedatives or tranquil* or neuroleptic* or psychiatric medication or depot or Benzo* or benzos or benzod* or non-benzo* or z-drug* or barbiturates* or Tricyclic Anti* or Olanzapine or Ziprasidone or Zopliclone or Zopidem or Zaleplon or Allobarbital or Alphenal or Brallobarbital or Pipotiazine or Zuclopenthixol or Levomepromazine or Amisulpride or Asenapine or Valium or Pericyazine or Clobazam or Clonazepam or ketazolam or Halazepam or loprazolam or Quinalbarb* or luminal or Librax or Butobarbitone or Amylobarbitone or Adinazolam or Mirtazapine or Bretazenil or Brotizaolam or Camazepam or Cinolazepam or Clotiazepam or Cloxazolam or Deslorazepam or Etizolam or Fludiazepam or Haloxazolam or Oxazolam or Nimetazepam or Nordazepam or Phenazepam or Pinazepam or Tetrazepam or Tofisopam or Quazepam or Lormetazepam or Trazadone or pregabalin).mp,ti,ab | **476066** |
| 2 | (Mental Disorders or schizophren* or psychosis or psychotic disorders or paranoi* or healthy volunteer* or healthy control* or OCD or PTSD).mp,ti,ab | **669776** |
| 3 | (Social Cogniti* or social perception or social knowledge or social competence or emotion recognition or emotion perception or affect recognition or affect perception or attribution bias or theory of mind or mentali* or mindblindness or mind-reading or social judgment or empath* or emotional intelligence or EI or facial recognition or facial affect or facial expression or face perception or FEIT or FERT or interpersonal perception or interpersonal interaction or hinting task or SCRT or MCCB or sat-mc or "reading in the minds eye" or AIHQ or BLERT or CANTAB).mp,ab,ti. | **108068** |
| 4 | 1 AND 2 AND 3 | **520** |
| 5 | exp anti-anxiety agents/ or exp antipsychotic agents/ | **188662** |
| 6 | (psychotropic or antipsychotic* or Hypnotic* or Sedative* or tranquil* or neuroleptic* or "psychiatric medication*" or depot or Benzo* or benzos or benzod* or non-benzo* or z-drug* or barbiturates* or Tricyclic Anti* or Olanzapine or Ziprasidone or Zopliclone or Zopidem or Zaleplon or Allobarbital or Alphenal or Brallobarbital or Pipotiazine or Zuclopenthixol or Levomepromazine or Amisulpride or Asenapine or Valium or Pericyazine or Clobazam or Clonazepam or ketazolam or Halazepam or loprazolam or Quinalbarb* or luminal or Librax or Butobarbitone or Amylobarbitone or Adinazolam or Mirtazapine or Bretazenil or Brotizaolam or Camazepam or Cinolazepam or Clotiazepam or Cloxazolam or Deslorazepam or Etizolam or Fludiazepam or Haloxazolam or Oxazolam or Nimetazepam or Nordazepam or Phenazepam or Pinazepam or Tetrazepam or (Tofisopam or Quazepam or Lormetazepam or Trazadone or pregabalin)).mp,ti,ab |  |
| 7 | 5 OR 6 | **572574** |
| 8 | exp Mental Disorders/ | **1318032** |
| 9 | (schizophren* or psychosis or "psychotic disorder*" or paranoi* or "healthy volunteer*" or "healthy control*" or OCD or PTSD).mp,ab,ti. | **499638** |
| 10 | 8 OR 9 | **1594954** |
| 11 | exp Social Perception/ | **24487** |
| 12 | ("social Cogniti*” or "social percept*" or "social knowledge" or "social competence" or "emotion recognition" or "emotion perception" or "affect recognition" or "affect percept*" or "attribution bias" or "theory of mind" or mentali* or mindblindness or mind-reading or "social judgment" or empath* or "emotional intelligence" or "facial recognition" or "facial affect" or "facial express*" or "face perception" or FEIT or FERT or "interpersonal percept*" or "interpersonal interaction" or "hinting task*" or SCRT or MCCB or sat-mc or "reading in the minds eye" or AIHQ or BLERT or CANTAB).mp,ab,ti. | **100832** |
| 13 | 11 OR 12 | **100832** |
| 14 | 7 AND 10 AND 13 | **719** |
| 15 | 14 NOT 4 | **214** |
| 16 | *filter inception-2019* | **207** |

**Example Search:**

Web of Science was searched on 30/12/2019 from inception to present dates.

| 1 | (TI=(anti-anxiety agents or antipsychotic agents or psychotropic or antipsychotic* or Hypnotics or Sedatives or tranquil* or neuroleptic* or psychiatric medication or depot or Benzo* or benzos or benzod* or non-benzo* or z-drug* or barbiturates* or Tricyclic Anti* or Olanzapine or Ziprasidone or Zopliclone or Zopidem or Zaleplon or Allobarbital or Alphenal or Brallobarbital or Pipotiazine or Zuclopenthixol or Levomepromazine or Amisulpride or Asenapine or Valium or Pericyazine or Clobazam or Clonazepam or ketazolam or Halazepam or loprazolam or Quinalbarb* or luminal or Librax or Butobarbitone or Amylobarbitone or Adinazolam or Mirtazapine or Bretazenil or Brotizaolam or Camazepam or Cinolazepam or Clotiazepam or Cloxazolam or Deslorazepam or Etizolam or Fludiazepam or Haloxazolam or Oxazolam or Nimetazepam or Nordazepam or Phenazepam or Pinazepam or Tetrazepam or Tofisopam or Quazepam or Lormetazepam or Trazadone or pregabalin)) OR AB=(anti-anxiety agents or antipsychotic agents or psychotropic or antipsychotic* or Hypnotics or Sedatives or tranquil* or neuroleptic* or psychiatric medication or depot or Benzo* or benzos or benzod* or non-benzo* or z-drug* or barbiturates* or Tricyclic Anti* or Olanzapine or Ziprasidone or Zopliclone or Zopidem or Zaleplon or Allobarbital or Alphenal or Brallobarbital or Pipotiazine or Zuclopenthixol or Levomepromazine or Amisulpride or Asenapine or Valium or Pericyazine or Clobazam or Clonazepam or ketazolam or Halazepam or loprazolam or Quinalbarb* or luminal or Librax or Butobarbitone or Amylobarbitone or Adinazolam or Mirtazapine or Bretazenil or Brotizaolam or Camazepam or Cinolazepam or Clotiazepam or Cloxazolam or Deslorazepam or Etizolam or Fludiazepam or Haloxazolam or Oxazolam or Nimetazepam or Nordazepam or Phenazepam or Pinazepam or Tetrazepam or Tofisopam or Quazepam or Lormetazepam or Trazadone or pregabalin) | **465,192** |
| --- | --- | --- |
| 2 | (TI=(Mental Disorders or schizophren* or psychosis or psychotic disorders or paranoi* or healthy volunteer* or healthy control* or bipolar or OCD or PTSD)) OR AB=(Mental Disorders or schizophren* or psychosis or psychotic disorders or paranoi* or healthy volunteer* or healthy control* or bipolar or OCD or PTSD) | **815,972** |
| 4 | (TI=(Social Cogniti* or social perception or social knowledge or social competence or emotion recognition or emotion perception or affect recognition or affect perception or attribution bias or theory of mind or mentali* or mindblindness or mind-reading or social judgment or empath* or emotional intelligence or EI or facial recognition or facial affect or facial expression or face perception or FEIT or FERT or interpersonal perception or interpersonal interaction or hinting task or SCRT or MCCB or sat-mc or "reading in the minds eye" or AIHQ or BLERT or CANTAB)) OR AB=(Social Cogniti* or social perception or social knowledge or social competence or emotion recognition or emotion perception or affect recognition or affect perception or attribution bias or theory of mind or mentali* or mindblindness or mind-reading or social judgment or empath* or emotional intelligence or EI or facial recognition or facial affect or facial expression or face perception or FEIT or FERT or interpersonal perception or interpersonal interaction or hinting task or SCRT or MCCB or sat-mc or "reading in the minds eye" or AIHQ or BLERT or CANTAB) | **532,873** |
| 5 | ((#3) AND #2) AND #1 | **1405** |
| 6 | *Filter inception-30/12/2019* | **1259** |
